# Placental AA/EPA Ratio Is Associated with Obesity Risk Parameters in the Offspring at 6 Years of Age

**DOI:** 10.3390/ijms241210087

**Published:** 2023-06-13

**Authors:** Ariadna Gómez-Vilarrubla, Berta Mas-Parés, Gemma Carreras-Badosa, Mariona Jové, Rebeca Berdún, Alexandra Bonmatí-Santané, Francis de Zegher, Lourdes Ibañez, Abel López-Bermejo, Judit Bassols

**Affiliations:** 1 Maternal-Fetal Metabolic Research Group, Girona Institute for Biomedical Research (IDIBGI), 17190 Salt, Spain; agomez@idibgi.org; 2 Pediatric Endocrinology Research Group, Girona Institute for Biomedical Research (IDIBGI), 17190 Salt, Spain; bmas@idibgi.org (B.M.-P.); gcarreras@idibgi.org (G.C.-B.); 3 Department of Experimental Medicine, University of Lleida-Biomedical Research Institute of Lleida (UdL-IRBLleida), 25008 Lleida, Spain; mariona.jove@udl.cat (M.J.); rebecaberdun@gmail.com (R.B.); 4 Department of Gynecology, Dr. Josep Trueta Hospital, 17007 Girona, Spain; alexandra.bonmati.santane@gmail.com; 5 Department of Development & Regeneration, University of Leuven, 3000 Leuven, Belgium; francis.dezegher@uzleuven.be; 6 Endocrinology, Pediatric Research Institute, Sant Joan de Déu Children’s Hospital, 08950 Esplugues de Llobregat, Spain; lourdes.ibanez@sjd.es; 7 CIBERDEM (Spanish Biomedical Research Centre in Diabetes and Associated Metabolic Disorders), ISCIII, 28029 Madrid, Spain; 8 Department of Pediatrics, Dr. Josep Trueta Hospital, 17007 Girona, Spain; 9 Department of Medical Sciences, University of Girona, 17003 Girona, Spain

**Keywords:** placenta, lipidomics, LC-PUFA, AA/EPA ratio, fatty acid transporters, visceral adiposity, fetal programming

## Abstract

During pregnancy, maternal polyunsaturated fatty acids (PUFA) are transferred to the fetus through the placenta by specific FA transporters (FATP). A higher perinatal exposure to n-6 over n-3 PUFA could be linked to excess fat mass and obesity development later in life. In this context, we aimed to assess the associations between long chain PUFAs (LC-PUFAs) (n-6, n-3, and n-6/n-3 ratios) measured in the placenta at term birth with obesity-related parameters in the offspring at 6 years of age and assess whether these associations are dependent on the placental relative expression of fatty acid transporters. As results, the PUFAn-6/PUFAn-3 ratio was 4/1, which scaled up to 15/1 when considering only the arachidonic acid/eicosapentaenoic acid ratio (AA/EPA ratio). Positive associations between the AA/EPA ratio and offspring’s obesity risk parameters were found with weight-SDS, BMI-SDS, percent fat mass-SDS, visceral fat, and HOMA-IR (r from 0.204 to 0.375; all *p* < 0.05). These associations were more noticeable in those subjects with higher expression of fatty acid transporters. Therefore, in conclusion, a higher placental AA/EPA ratio is positively associated with offspring’s visceral adiposity and obesity risk parameters, which become more apparent in subjects with higher expressions of placental FATPs. Our results support the potential role of n-6 and n-3 LC-PUFA in the fetal programming of obesity risk in childhood. For the present study, 113 healthy pregnant women were recruited during the first trimester of pregnancy and their offspring were followed up at 6 years of age. The fatty acid profiles and the expression of fatty acid transporters (FATP1 and FATP4) were analyzed from placental samples at birth. Associations between LC-PUFA (n-6, n-3, and n-6/n-3 ratios) and obesity risk parameters (weight, body mass index (BMI), percent fat mass, visceral fat, and homeostatic model assessment of insulin resistance (HOMA-IR)) in the offspring at 6 years of age were examined.

## 1. Introduction

Polyunsaturated fatty acids (PUFAs) are vital macronutrients that are not only required as energy sources, but are also indispensable for their structural and metabolic functions [1], as well as for their involvement in cell signalling processes [2] and inflammation [3]. Alpha-linolenic acid (ALA) and linoleic acid (LA) are essential fatty acids (EFAs) and precursors of the omega 3 and omega 6 series, respectively, that cannot be synthesized by the human body and must be acquired through ingestion [4]. Long chain PUFAs (LC-PUFAs), such as eicosapentaenoic acid (EPA; 20:5n-3), docosahexaenoic acid (DHA; 22:6n-3), and arachidonic acid (AA; 20:4n-6), are synthesized from ALA and LA, though they can also be acquired from the diet. These LC-PUFAs are lipid mediators of critical importance in early development stages [5,6] and throughout the life course [7]. AA and EPA compete for the same conversion enzymes and are involved in similar processes [8] which give rise to derivatives with opposing health effects [9]. The modern dietary transition towards an excessive n-6 consumption with respect to n-3 PUFA has led to a shift in the LA/ALA ratio from 1/1 up to 10–30/1 [10], which has been linked to the pathogenesis of several metabolic diseases such as atherosclerosis, diabetes, and obesity [11].

Maternal PUFA n-3 and n-6 have been linked to placental function [12] and pregnancy [13]. The placenta is a transitory organ which harbours the growing fetus while enabling the exchange of oxygen, hormones, nutrients, and waste products between the maternal and fetal circulatory systems [14]. Given the low capability of the placenta to convert EFAs to LC-PUFAs, to meet the fetal demand both EFAs and LC-PUFAs are mainly supplied by the mother through the placenta [12]. The placental supply of LC-PUFA can be derived from maternal adipose tissue and maternal dietary fatty acid intake [15], reaching the fetus both by simple diffusion or by facilitated transport through fatty acid transporters (FATPs) [16]. Placental lipid transport and metabolism affect the quantity and quality of lipids delivered to the fetus [17]. FATP1 and FATP4 have been shown to be important in cellular LC-PUFA handling and in metabolism regulation in skeletal muscle [18], while they have also been reported to be significantly increased in the placentas of overweight pregnant women [19].

To date, a high n-6/n-3 PUFA ratio in umbilical cord plasma [20] and human breast milk [21] during pregnancy and lactation has been associated with increased children’s adiposity, observed as early as four months of age. Despite the fact that the placenta has been proposed as a surrogate tissue for the effects of maternal alterations on the fetus during pregnancy [22], to our knowledge, no research on the placental n-6/n-3 PUFA ratio and its potential effects on the offspring’s health outcomes has been conducted so far.

In this context, we aimed to assess the associations between placental LC-PUFA n-6, n-3, and n-6/n-3 ratios with obesity-related parameters in offspring at six years of age, and to assess whether they were related to the relative expression of placental Ʃ FATPs.

## 2. Results

### 2.1. Descriptive Characteristics of the Mother-Infant Pairs

Clinical and anthropometric characteristics of the mother-newborn pairs and the offspring’s follow-up are presented in Supplementary Table S1. A total of 113 mothers with an average age of 30.8 ± 4.2 years were enrolled in the study, and 73% of these returned for the follow-up visit of their children (n = 82; 45 boys and 37 girls) at an average age of 5.9 ± 0.9. Mothers had an average pre-pregnancy BMI of 24.9 ± 4.7 kg/m^2^ and gained 14.4 ± 4.6 kg during gestation. Children at the time of the 6-year follow-up showed z-score values of 0.16 ± 1.22 and 0.13 ± 1.06 for weight and BMI, respectively. Maternal anthropometric data did not present statistically significant differences when comparing mothers whose children attended the 6-year follow-up visit and those who did not.

No associations between children’s physical activity or nutritional status with visceral adiposity and obesity risk parameters were found.

### 2.2. Placental Fatty Acid Profile

**Figure 1:**
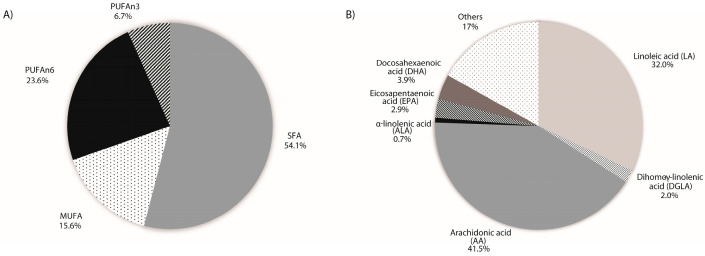
(**A**) Representation of the abundance of the calculated fatty acids groups. SFA (saturated fatty acids), MUFA (monounsaturated fatty acids), PUFA n-6 (polyunsaturated fatty acids (n-6)), PUFA n-3 (polyunsaturated fatty acids (n-3)). (**B**) Representation of the abundance of the most relevant n-6 and n-3 polyunsaturated fatty acids.

Table 1 compiles the percentages of relative abundance of each fatty acid analyzed from the 113 placental samples. Saturated fatty acids represented around half of the total placental fatty acids fraction (54.1%) (Figure 1A). The other half of abundance corresponded to MUFA (15.6%), PUFAn-6 (23.6%), and PUFAn-3 (6.7%) (Figure 1A). Within the PUFA fatty acids fraction (PUFAn-6 + PUFAn-3), it is worth mentioning that arachidonic acid (AA, 20:4n-6) represented 41.5% of the PUFA abundance (Figure 1B). On the other hand, eicosapentaenoic acid (EPA, 20:5n-3) and docosahexaenoic acid (DHA, 22:6n-3) accounted for 2.9% and 3.9% of the PUFA abundance, respectively (Figure 1B). Finally, the n-6/n-3 PUFA ratio was 4/1, while the ratio of AA to EPA was 15/1 (Table 1).

### 2.3. Placental Long Chain Polyunsaturated Fatty Acid (LC-PUFA) Abundance and Offspring Anthropometric and Metabolic Traits

AA and EPA, which stand out for their functional roles in growth and development, and metabolism, respectively, as well as the AA/EPA and AA/EPA + DHA ratios, were further analysed for correlations with offspring adiposity and metabolic parameters (Table 2).

**Figure 2:**
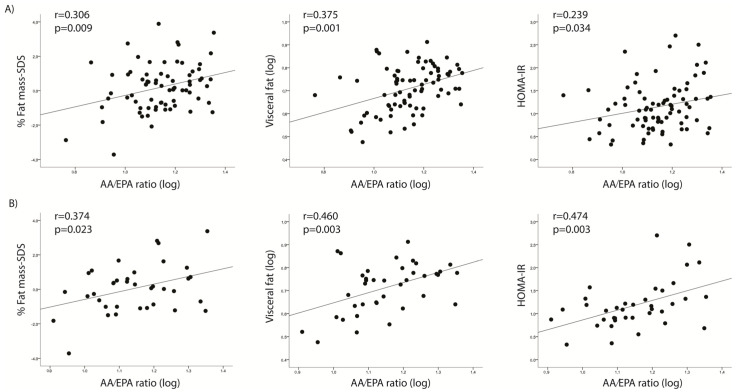
(**A**) Pearson’s correlation between the AA/EPA ratio and the metabolic parameters of the offspring in (**A**) the whole population (N = 82) and (**B**) the group with higher relative expression of fatty acid transporters (FATPs) (Ʃ FATPs > 50th centile) (N = 40).

In the n-6 series, AA displayed significant positive associations with offspring visceral fat (r = 0.225, *p* = 0.04). Moreover, positive associations were identified between the AA/EPA ratio and weight-SDS, BMI-SDS, percent fat mass-SDS, visceral fat, and HOMA-IR (r between 0.202 and 0.383; *p* < 0.05) (Table 2; Figure 2A). The AA/EPA + DHA ratio also showed positive associations with visceral fat (r = 0.254, *p* = 0.03).

Most of these associations remained significant in multiple regression analysis after adjusting for maternal age, gestational weight gain (GWG), gestational age at birth, birth weight, and age, sex, nutrition score, and physical activity at follow-up. (Table 2).

### 2.4. Placental Fatty Acid Transporter Relative Expression

Table 3 presents data on the placental relative gene expression of FATP1 and FATP4 and the sum of the expression of FATP1 and FATP4 (Ʃ FATPs). The functionally relevant fatty acids, AA, EPA, PUFAn-6, and PUFAn-3, together with the PUFAn-6/PUFAn-3 ratio were positively associated with Ʃ FATPs (*p* < 0.05) (Supplementary Table S2). These associations maintained their statistical significance after adjusting for potential confounding variables.

### 2.5. Placental Ʃ FATP Relative Expression Analysis

Given that higher Ʃ FATP expression is associated with higher amounts of LC-PUFA of the n-6 and n-3 series and the PUFAn-6/PUFAn-3 ratio, we further analyzed the associations between LC-PUFA and the studied offspring variables in subgroups according to the median relative expression of Ʃ FATPs (50th centile) (Table 4). Mothers included in the group of the highest 50th centile (Ʃ FATPs > 50th centile) presented higher GWG (Supplementary Table S3).

Within the group of mothers in the highest 50th centile (Ʃ FATPs > 50th centile) the percent fat-mass-SDS was positively associated with both LC-PUFA from the n-6 series (AA and PUFAn-6) and with LC-PUFA from the n-3 series (DHA and PUFAn-3), while visceral fat and HOMA-IR were positively associated with the n-6 series (AA and PUFAn-6, and AA, respectively) (Table 4). The percent fat-mass-SDS, visceral fat, and HOMA-IR were positively associated with the AA/EPA ratio (Table 4; Figure 2B) (r between 0.374 and 0.474; *p* < 0.05). Most of these associations remained significant after adjusting for potential confounding variables (maternal age, GWG, newborn gestational age, birth weight, and age and sex at follow-up for the variables related to the 6-year-old children) in multiple regression analysis (Table 4).

## 3. Discussion

We outline for the first time the associations between placental n-6 and n-3 LC-PUFAs and the n-6/n-3 PUFA ratio with offspring visceral adiposity and obesity risk at age six in children from the general population.

In our study, SFA accounted for more than half of the placental fatty acid abundance, while PUFAs only represented around 30% of the abundance. Yamazaki I et al. found that Japanese placentas contained 38–39% SFAs and 46–48% PUFAs. The differences between both results might be due to dietary differences, as Japanese society is known to consume more PUFA-rich fish and seafood [23].

From another perspective, our findings fit with the observed progressive decrease in DHA levels in maternal plasma and red blood cells (RBC) during normal pregnancies [24], and the higher n-3/n-6 PUFA ratio found in infants’ RBC compared to lactating mothers’ [25]. The observed low placental PUFA abundance might be due to a high transfer to the fetus, which mainly occurs in late pregnancy, specifically during the 3rd trimester of gestation, when there is a high demand for PUFAs, which must be supplied by the mother [26]. Thus, fat deposition in the fetus increases exponentially with gestational age and reaches its maximal rate of accretion just before term [16]. Although there is a relative small net loss of maternal adipose tissue during the period of maximal fat deposition in the fetus, the very high fasting TG levels in the mother suggest a high maternal body fat turn-over compared to the non-pregnant state [27].

Despite the low abundance of placental PUFAs found in this study, we found the PUFAn-6/PUFAn-3 ratio to be in a proportion of 4/1, which scaled up to 15/1 when focusing on the AA/EPA ratio. The observed excess of n-6 PUFAs over n-3 PUFAs in the present study would match with the excessive consumption of n-6 PUFAs described in the modern Western diet, which in turn has been linked to an increased risk of obesity and other metabolic disorders [28,29]. Although our study lacks data on maternal food intake, results of our own assessed in a similar cohort of 384 pregnant women (recruited in the same medical region and with the same inclusion and exclusion criteria as in the present study) not yet published, showed an intake ratio of 8/1 for n-6 versus n-3 PUFAs. The food intake was estimated by using the same food frequency questionnaire (FFQ) as Vioque et al. [30], who found a similar n-6 versus n-3 proportion of 8/1 in a comparable cohort of 740 pregnant women from Valencia (Spain). This evidence could point out that the observed PUFA ratios are in line with the current Western diet and that the increased metabolic risk is implied with the n-6/n-3 proportion despite the total PUFA presence.

Obesity risk in childhood and later life might be influenced by early life inputs. In this sense, the higher n-6/n-3 PUFA ratio in maternal diet, maternal blood, and cord blood was previously associated with higher adiposity at 3 years of age [20]. In the same way, Vaidya et al. found that breast milk with a higher n-6/n-3 PUFA ratio had higher pro-inflammatory cytokines that altered the adipose tissue metabolism, predisposing newborns to a higher risk of obesity in later life [31]. The authors also pointed out the upregulation of lipogenic gene expression in preadipocytes treated with breast milk from non-obese women with high n-6/n-3 PUFA ratios [31]. Similarly, in our results, the placental AA/EPA ratio was longitudinally associated with weight-SDS, BMI-SDS, percent fat mass-SDS, visceral fat, and HOMA-IR in the six-year-old offspring, regardless of the effect of potential confounding variables.

The various correlations found between the placental AA/EPA ratio and risk parameters for visceral adiposity and obesity in our six-year-old children support the idea that the relative abundance of n-6 over n-3 PUFAs may have a stronger influence on the adipose tissue than n-6 PUFA abundance itself [20,32]. It is noteworthy that n-6 and n-3 PUFAs compete for the fatty acid desaturase and elongase enzymes [33] and the current n-6/n-3 ratio disproportion, related to the modern Western diet, might lead to a higher proportion of n-6 derivatives, such as AA, with respect to n-3 derivatives, such as EPA [34]. In this context, Aparicio et al. found that fish and seafood consumption during pregnancy increased EPA concentration and reduced the maternal serum n-6/n-3 PUFA ratio and AA/EPA ratio in the first and third trimester of gestation [35]. Similarly, n-3 PUFA supplementation during pregnancy has been shown to have beneficial effects on neonatal health [36].

EPA is a key anti-inflammatory LC-PUFA [37] capable of lowering serum TG and non-HDL-C [38]. The administration of EPA increases the EPA/AA ratio and has been shown to reduce the risk of cardiovascular disease and its progression [39]. Low serum EPA/AA ratios were significantly associated with an increased risk of coronary heart disease [40,41]. On the other hand, AA has been related to adipocyte growth and differentiation [42]. This finding could explain the association found between the placental AA and the higher visceral fat in the six-year-old children in our study.

Maternal adipose tissue together with maternal dietary supply are crucial fetal sources of LC-PUFAs [16]. While in utero, the transference of nutrients across the placenta is the single source of nutrition, and the n-6 and n-3 PUFAs have to cross the placenta preferentially through fatty acid transporters [43]. FATP1 and FATP4 are the only transporters with acyl-CoA-synthetase activity that prevent PUFA efflux [44]. Song et al. recently reported a significant increase in FATP4 placental mRNA expression among other genes involved in lipid metabolism in overweight mothers [19], while others reported lower FATP4 protein expression in placentas from women with high pre-pregnancy BMI or gestational diabetes mellitus [45]. Regarding FATP1, it was reported to be up-regulated under pregnancies with high-fat diets, while it was downregulated with a standard diet during pregnancy in mice [46]. In our study, AA, DHA and the AA/EPA ratio associations with the offspring’s visceral adiposity and obesity risk parameters were predominant in the group of higher FATPs relative expression (Ʃ FATPs > 50th centile), in line with the findings of Larqué et al., in which FATP1 and FATP4 placental expressions were found to be involved in the placental transfer of LC-PUFAs [47]. Additionally, those mothers within the higher 50th centile (Ʃ FATPs > 50th centile) presented higher GWG. As a whole, we may speculate that the greater LC-PUFA transfer related to higher FATP expression, which in turn may have a greater metabolic effect on the offspring’s health outcomes, might be subject to a higher energy intake resulting in a higher maternal GWG.

Within the highest FATP 50th centile (Ʃ FATPs > 50th centile), the percent fat mass-SDS was positively associated both with PUFAn-6 and PUFAn-3. Despite the fact that omega 3 PUFAs are attributed an anti-inflammatory and protective role [37], the positive associations found between the LC-PUFAn-3 and the percent fat mass-SDS might be related to the activation of brown adipose tissue or the induction of beige adipocytes in white adipose tissue [48].

We acknowledge some strengths and limitations in our study. One of the major strengths is its longitudinal design, which has allowed exploration of the associations of n-6 and n-3 LC-PUFAs and fatty acid transporters with children’s metabolic outcomes within 73% of the subjects enrolled in the study. As a major limitation, data on maternal food intake and nutritional status throughout pregnancy were not available, nor were maternal and offspring n-6 and n-3 plasma abundances, which would have been of interest to have a broader overview of the lipid profile during pregnancy and in the offspring. Studies using a larger number of individuals are warranted to confirm the associations found herein.

In conclusion, the placental AA/EPA ratio was associated with visceral adiposity and obesity risk parameters. Those associations were more pronounced in subjects with higher Ʃ FATP placental relative expression. Our results support the potential role of n-6 and n-3 LC-PUFAs in fetal programming of obesity risk in childhood. 

## 4. Materials and Methods

### 4.1. Study Population and Ethics

This is a prospective longitudinal study of 113 pregnant Caucasian women and their newborns that were included in a mother–children cohort.

Pregnant women were included during the first trimester of pregnancy from among women seen within a primary care setting in Girona (Spain) [49], and children were followed-up with at six years of age.

The inclusion period was between 1 January 2007 and 31 December 2010 and the follow-up with the children was between 1 January 2013 and 31 December 2017.

Caucasian women with uncomplicated pregnancies delivering healthy infants at term (37–42 weeks) from singleton pregnancies were included, irrespective of the type of delivery. The exclusion criteria were hypertension, pre-eclampsia, gestational diabetes or pre-existing type 1 diabetes mellitus, as well as multiple pregnancies, fetal growth restriction, malformations, asphyxia, or any other obstetrical complications. Assisted reproductive technology, smoking, and drug or alcohol abuse during pregnancy were also considered exclusion criteria.

The protocol was approved by the Ethics Committee of the Institutional Review Board of Dr. Josep Trueta Hospital (Girona), and informed written consent was obtained from all the subjects enrolled in the study.

### 4.2. Maternal Anthropometric Assessments

Maternal age at conception, pre-gestational weight, and maternal height were measured in the first antenatal visit (between 6 and 9 weeks of gestation). Body-mass index (BMI) was calculated as weight divided by height squared (kg/m^2^). Maternal weight was also assessed at the last visit before delivery (between 36 and 42 weeks of gestation; pre-delivery weight). GWGwas calculated from the deduction of the pre-gestational weight from the pre-delivery weight.

### 4.3. Infant Anthropometric and Clinical Assessments at Delivery

After delivery, newborns’ weight and length were measured using a calibrated scale and a measuring board. Gestational age- and sex-adjusted z-scores (SDS) for birth weight and length were calculated using regional norms [50]. The Ponderal index was calculated as weight (g) multiplied by 100 and divided by length to the third power (cm^3^).

### 4.4. Infant Anthropometric and Clinical Assessments at Six Year Follow-Up

All participants were invited to the follow-up visit at six years of age. Out of the 113 newborns, 82 children agreed to continue participating in the study and attended the follow-up visit at 6 years of age, and 31 failed to continue and hence such cases were not considered for further analysis in the study (Supplementary Figure S1). Weight was measured on a calibrated scale wearing light clothes, and height was measured barefooted with a Harpenden stadiometer. BMI was calculated, and weight, height, and BMI-SDS scores were derived as mentioned above [50]. Waist and hip circumference were measured in the supine position at the umbilical region and at the widest part at the level of the greater trochanters, respectively. Fat mass was assessed by bioelectric impedance (Hydra Bioimpedance Analyzer 4200; Xitron Technologies; San Diego, CA, USA). 

Venous blood sampling was performed in the morning under fasting conditions. Serum glucose, insulin, total serum triglyceride (TG), cholesterol, and high-density lipoprotein (HDL) were assayed using standardized procedures [51]. Fasting insulin sensitivity was estimated from fasting insulin and glucose levels using the formula HOMA-IR = (fasting insulin in mU/L) × (fasting glucose in mg/dL)/405). 

High-resolution ultrasound was used to estimate visceral fat volume as described by Hirooka et al. [52] after taking the measurements using high-resolution ultrasonography (MyLab^TM^25, Esaote, Firenze, Italy) in a transverse abdominal scan with a convex 3–3.5 MHz transducer. Averages of three measurements were used in the study. All measurements were performed by the same operator who was blinded to treatment allocation. The intra-subject coefficient variation was less than 6%.

In the same follow-up visit, infants’ physical activity and nutritional status were assessed using questionnaires (paper form) that were completed by the parents. Adapted versions of the standardized questionnaires from the EnKid study and KIDMED were used [53,54]. Nutritional status was evaluated through a nutrition score (which ranges from −4 to 12) and in which higher values mean higher adherence to a healthy Mediterranean diet [53]. Physical activity was evaluated according to duration (hours) of daily activities (at school, extracurricular activities, at home, and also weekend activity) and intensity was calculated from the duration using metabolic equivalents to tasks (METS) [55]. 

### 4.5. Placental Sample Collection

After delivery, placentas were weighed and immediately processed. Three biopsies of 1 cm^3^ were dissected from the inner surface of the placenta (maternal side) from randomly selected lobes. Afterwards, the decidua layer was removed and the placental samples were washed with physiological saline buffer and immediately frozen at −80 °C until analyzed [56]. Specifically, from the same sample, one aliquot was used for the analysis of the placental fatty acid profile and one other aliquot was used for mRNA expression analysis of the fatty acid transporters.

### 4.6. Placental Fatty Acid Profile

Percentages of total fatty acids from placental lipids (including glycerolipids, phospholipids, sphingolipids, sterol lipids, and free fatty acids) were analyzed as fatty acid methyl ester derivatives (FAMEs) by gas chromatography (GC) as previously described [57]. Briefly, lipids from 500 mg of placental samples were extracted with chloroform/methanol (2:1, *v*/*v*) in the presence of 0.01% butylated hydroxytoluene. The fatty acids were trans-esterified and the resulting fatty acid methyl esters were extracted. A total amount of 4 μL were used for GC analysis. Separation was performed with a DBWAX capillary column (30 m × 0.25 mm × 0.20 μm) in a GC System 7890A with a Series Injector 7683B and an FID detector (Agilent Technologies, Barcelona, Spain). Identification of fatty acid methyl esters was made by comparison with authentic standards (Larodan Fine Chemicals, Malmö, Sweden). Results are expressed as mol percentage (mol %) [56].

The following fatty acids groups were determined: saturated fatty acids (SFA); monounsaturated fatty acids (MUFA); polyunsaturated fatty acids (PUFA) from n-6 and n-3 series (PUFAn-6 and PUFAn-3, respectively); and the following ratios were also calculated: LA/ALA, AA/EPA, AA/DHA, AA/EPA + DHA, and the n-6/n-3 PUFA ratio as the quotient between all the PUFA n-6s divided by all the PUFA n-3s. 

### 4.7. Gene Expression Analysis

Placental RNA isolation was performed using the RNeasy mini kit (QIAGEN) and it was retrotranscribed using the High-Capacity cDNA Reverse Transcription Kit (Applied Biosystems). FATP1 and FATP4 gene expressions were assessed with the commercially available TaqMan assays (Thermofisher Scientific, Walthan, MA, USA) Hs01587917_m1 (FATP1) and Hs00192700_m1 (FATP4), using the TaqMan technology in a LightCycler 480 Real-Time PCR System (Roche Diagnostics, Rotkreuz, Switzerland). Relative gene expression levels were calculated according to the 2^−ΔCt^ method, using the average values obtained by endogenous controls for placental samples [TaqMan assays SDHA (Hs00188166_m1) and TBP (Hs00427620_m1)]. 

### 4.8. Data Analysis

Data analyses were performed with the statistical software SPSS, version 22.0 (SPSS, Chicago, IL, USA). Results are expressed as mean ± standard deviation (SD). Variables without normal distribution were logarithmically transformed. Associations between variables were assessed through bivariate Pearson correlation analyses. Independent associations between variables were assessed by multiple regression analyses adjusting for potential confounding variables (maternal age, GWG, gestational age at birth, birth weight, and age, sex, nutrition score, and physical activity at follow-up). The significance level was set to a *p* value < 0.05. 

## Figures and Tables

**Table 1:** Relative abundance percentage of the analyzed fatty acids and calculated fatty acid ratios of interest.

Fatty Acid Profile		All Population
		N = 113
		Mean ± SD
**SFA (mol %)**		
14:0	Myristic acid	1.59 ± 0.46
16:0	Palmitic acid	33.03 ± 2.50
18:0	Stearic acid	17.27 ± 1.50
20:0	Arachidic acid	0.32 ± 0.08
22:0	Behenic acid	1.33 ± 0.92
24:0	Lignoceric acid	0.53 ± 0.47
SFA		54.07 ± 3.99
**MUFA (mol %)**		
16:1n-7	Palmitoleic acid	0.50 ± 0.15
18:1n-7	Vaccenic acid	2.00 ± 0.35
18:1n-9	Oleic acid	12.16 ± 1.55
20:1n-9	Eicosenoic acid	0.26 ± 0.06
22:1n-9	Erucic acid	0.67 ± 0.60
MUFA		15.59 ± 2.17
**PUFA n-6 series (mol %)**		
18:2n-6	Linoleic acid (LA)	9.71 ± 1.39
20:2n-6	Eicosadienoic acid	0.10 ± 0.07
20:3n-6	Dihomo-γ-linolenic acid (DGLA)	0.61 ± 0.09
20:4n-6	Arachidonic acid (AA)	12.59 ± 3.74
22:4n-6	Adrenic acid	0.51 ± 0.17
22:5n-6	Osbond acid	0.10 ± 0.06
PUFAn-6		23.62 ± 4.56
**PUFA n-3 series (mol %)**		
18:3n-3	α-linolenic acid (ALA)	0.22 ± 0.07
18:4n-3	Stearidonic acid	0.65 ± 0.58
20:3n-3	Eicosatrienoic acid	3.54 ± 1.16
20:5n-3	Eicosapentaenoic acid (EPA)	0.89 ± 0.25
22:5n-3	Docosapentaenoic acid (DPA)	0.25 ± 0.08
22:6n-3	Docosahexaenoic acid (DHA)	1.18 ± 0.52
PUFAn-3		6.72 ± 1.26
**n-6/n-3 PUFA RATIOS**		
AA/EPA		14.59 ± 4.05
AA/DHA		11.80 ± 4.03
AA/EPA + DHA		6.31 ± 1.58
PUFAn-6/PUFAn-3		3.55 ± 0.57

Data are shown as mean ± SD. SFA: saturated fatty acids; MUFA: monounsaturated fatty acids; PUFAn-6: polyunsaturated fatty acids (omega 6); PUFAn-3: polyunsaturated fatty acids (omega 3).

**Table 2:** Pearson correlation for the studied polyunsaturated fatty acids from the placenta and its ratios of interest with selected anthropometric and metabolic variables in the offspring at 6 years.

Follow-Up N = 82	Weight-SDS	BMI- SDS	% Fat Mass-SDS	Visceral Fat	HOMA-IR
**n-6 series**					
AA (20:4n-6)	0.110	0.062	0.126	0.225 *	0.156
PUFAn-6	0.070	0.028	0.098	0.203	0.143
**n-3 series**					
EPA (20:5n-3)	−0.113	−0.129	−0.159	−0.105	−0.049
DHA (22:6n-3)	0.012	−0.039	0.149	0.123	0.168
PUFAn-3	0.055	0.039	0.190	0.153	0.158
**n-6/n-3 ratios**					
AA/EPA	0.245 *	0.202 *	0.306 **	0.383 **	0.239 *
AA/DHA	0.100	0.115	−0.065	0.080	−0.053
AA/EPA + DHA	0.183	0.170	0.119	0.254 *	0.085
PUFAn-6/PUFAn-3	0.030	−0.007	−0.082	0.094	0.008

Data are shown as Pearson correlation coefficients. * *p* < 0.05 and ** *p* < 0.01. SDS: standard deviation score; BMI: body mass index; HOMA-IR: homeostatic model assessment of insulin resistance; AA: arachidonic acid; PUFAn-6: polyunsaturated fatty acids (omega 6); EPA: eicosapentaenoic acid; DHA: docosahexaenoic acid; PUFAn-3: polyunsaturated fatty acids (omega 3). Significant associations after adjustment for maternal age, gestational weight gain, gestational age at birth, birth weight, and age, sex, nutrition score, and physical activity at follow-up for the variables related to the 6-year-old children in multiple regression analysis are shown in bold.

**Table 3:** Placental fatty acid transporter relative expression (2^−Δct^).

Placental Fatty Acid Transporters (2^−ΔCt^)	All Population
	N = 113
	Mean ± SD
FATP1	0.19 ± 0.19
FATP4	0.33 ± 0.20
Ʃ FATPs	0.52 ± 0.32

Data are shown as mean ± SD. FATP: fatty acid transporter; Ʃ FATPs: sum of FATP1 and FATP4 expression.

**Table 4:** Pearson correlation for the studied polyunsaturated fatty acids from the placenta and its ratios of interest with selected anthropometric and metabolic variables in the offspring at 6 years, grouped considering the median expression of fatty acid transporters (FATPs) (Ʃ FATPs 50th centile).

Ʃ FATP1 + FATP4 < 50th Centile N = 42	Weight-SDS	BMI- SDS	% Fat Mass-SDS	Visceral Fat	HOMA-IR
**n-6 series**					
AA (20:4n-6)	0.173	0.148	0.113	0.196	0.038
PUFAn-6	0.097	0.089	0.038	0.147	0.041
**n-3 series**					
EPA (20:5n-3)	−0.153	−0.127	−0.348 *	−0.327 *	−0.057
DHA (22:6n-3)	−0.208	−0.095	0.005	−0.007	0.088
PUFAn-3	0.057	0.063	0.195	0.102	0.009
**n-6/n-3 ratios**					
AA/EPA	0.230	0.195	0.255	0.321 *	0.056
AA/DHA	0.273	0.181	0.092	0.165	−0.025
AA/EPA + DHA	0.256	0.189	0.170	0.248	0.016
PUFAn-6/PUFAn-3	0.062	0.049	−0.091	0.078	0.037
**Ʃ FATP1 + FATP4 > 50th centile** **N = 40**	**Weight-SDS**	**BMI-** **SDS**	**% Fat Mass-SDS**	**Visceral fat**	**HOMA-IR**
**n-6 series**					
AA (20:4n-6)	0.261	0.243	0.430 **	0.367 *	0.355 *
PUFAn-6	0.228	0.208	0.421 **	0.351 *	0.282
**n-3 series**					
EPA (20:5n-3)	−0.070	−0.034	−0.007	−0.161	−0.242
DHA (22:6n-3)	0.223	0.131	0.409 *	0.209	0.227
PUFAn-3	0.155	0.183	0.361 *	0.202	0.265
**n-6/n-3 ratios**					
AA/EPA	0.281	0.234	0.374 *	0.460 **	0.474 **
AA/DHA	−0.124	−0.006	−0.265	−0.001	−0.076
AA/EPA + DHA	0.070	0.126	0.023	0.317 *	0.245
PUFAn-6/PUFAn-3	0.030	−0.024	−0.030	0.101	−0.066

Data are shown as Pearson correlation coefficients. * *p* < 0.05 and ** *p* < 0.01. SDS: standard deviation score; BMI: body mass index; HOMA-IR: homeostatic model assessment of insulin resistance; AA: arachidonic acid; PUFAn-6: polyunsaturated fatty acids (omega 6); EPA: eicosapentaenoic acid; DHA: docosahexaenoic acid; PUFAn-3: polyunsaturated fatty acids (omega 3). Significant associations after adjustment for maternal age, gestational weight gain, gestational age at birth, birth weight, and age and sex at follow-up for the variables related to the 6-year-old children in multiple regression analysis are shown in bold.

## Data Availability

Data described in the manuscript, code book, and analytic code will be made available upon request to the corresponding author [JB].

## Supplementary Materials

The following supporting information can be downloaded at: https://www.mdpi.com/article/10.3390/ijms241210087/s1.

## Abbreviations

EFA	essential fatty acid
FATP	fatty acid transporter
GWG	gestational weight gain
SDS	standard deviation score
