# Supplementary material for: Placental AA/EPA Ratio Is Associated with Obesity Risk Parameters in the Offspring at 6 Years of Age
Source: Int J Mol Sci. 2023 Jun 13;24(12):10087. doi: 10.3390/ijms241210087 (PMC10298678; doi:10.3390/ijms241210087)
Supplement: Supplementary file 1 [file ijms-24-10087-s001.zip › Supplementary data.pdf]

Supplementary Table S1. Clinical and anthropometric assessments in the studied subjects.

	**COHORT**
**Mother**	**N=113**
Age at conception (years)	30.8±4.2
Primiparous (%)	54.9
Pre-gestational weight (kg)	66.97±13.88
Pre-gestational BMI (kg/m^2)	24.92±4.72
Height (cm)	163.83±6.16
Gestational weight gain (kg)	14.4±4.6
**Newborn**	
Sex (%F)	44.2
Placental weight (kg)	603.50±110.77
Weight (g)	3318.81±283.40
Weight-SDS	0.03±0.66
Length (cm)	49.58±1.55
Length-SDS	-0.199±0.905
Ponderal index (g/cm^3)*100	2.73±0.23
**Follow-up (6 years)**	
**Anthropometry**	**N= 82**
Gender (%F)	32.7
Age (years)	5.9±0.9
Weight (kg)	22.50±5.07
Weight-SDS	0.16±1.22
Height (cm)	115.86±7.86
Height-SDS	0.12±1.21
Hip (cm)	59.97±8.11
Waist (cm)	56.69±7.25
BMI (kg/m^2)	16.62±2.25
BMI-SDS	0.13±1.06
**Metabolic parameters**	
Fat mass (kg)	5.58±2.95
Fat mass-SDS	0.45±1.60
Fat mass (%)	23.59±8.16
% Fat mass-SDS	0.20±1.38
Visceral fat (cm^2)	5.24±1.15
Glucose (mg/dl)	82.91±7.43
Insulin (mlU/L)	5.51±2.24
HOMA-IR	1.15±0.52
Triglycerides (mg/dl)	50.49±15.62
Total Cholesterol (mg/dl)	163.87±26.35
HDL cholesterol (mg/dl)	55.37±10.86

Data are shown as mean ± SD. BMI: body-mass index; SDS: standard deviation score; HOMA-IR: homeostatic model assessment of insulin resistance; HDL: high-density lipoprotein.

Supplementary Table S2. Pearson correlation for the studied placental long chain polyunsaturated fatty acids (LC-PUFA) and its ratios with the sum of the studied placental lipid transporters (Σ *FATP*s).

**PLACENTAL LIPID TRANSPORTERS N=113**	**Σ *FATP*s**
**n-6 series**	
AA (20:4n-6)	**0.651****
PUFAn-6	**0.623****
**n-3 series**	
EPA (20:5n-3)	**0.617****
DHA (22:6n-3)	**0.636****
PUFAn-3	**0.532****
**n-6:n-3 ratios**	
AA/EPA	0.183
AA/DHA	-0.192
AA/EPA+DHA	-0.069
PUFAn-6/PUFAn-3	**0.205***

Data are shown as Pearson correlation coefficients. * p<0.05 and ** p<0.01. FATP: fatty acid transporter. AA: arachidonic acid; PUFAn-6: polyunsaturated fatty acids omega 6; EPA: eicosapentaenoic acid; DHA: docosahexaenoic acid; PUFAn-3: polyunsaturated fatty acids omega 3. Significant associations after the adjustment for maternal age, gestational weight gain, gestational age at birth, birth weight and sex in multiple regression analysis are shown in bold.

Supplementary Table S3. Comparison of the clinical and anthropometric assessments in the studied subjects between transporters groups (50th centile).

	**Σ *FATP*s < 50th centile**<br>**N=42**	**Σ *FATP*s > 50th centile**<br>**N=40**	
**Mother**			**p-value**
Age at conception (years)	31.5±3.6	30.6±4.4	Ns
Pre-gestational weight (kg)	65.36±13.69	68.39±14.68	Ns
Pre-gestational BMI (kg/m^2)	24.48±4.09	25.40±5.32	Ns
Height (cm)	163.06±6.14	164.1±6.08	Ns
Gestational weight gain (kg)	12.0±4.1	15.9±4.1	<0.001

Supplementary Figure S1: Flow chart of the participants recruited and followed-up.
