# Supplementary figures and images for: Placental AA/EPA Ratio Is Associated with Obesity Risk Parameters in the Offspring at 6 Years of Age
Source: Int J Mol Sci. 2023 Jun 13;24(12):10087. doi: 10.3390/ijms241210087 (PMC10298678; doi:10.3390/ijms241210087)

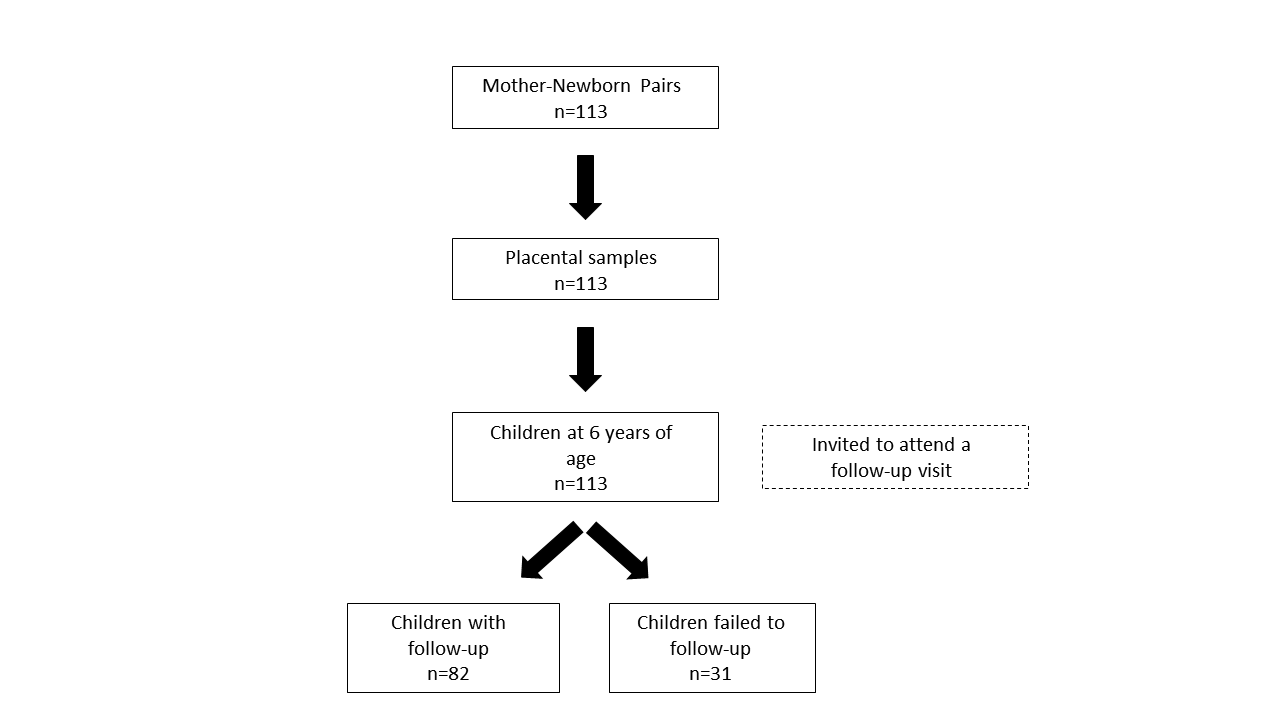

Supplement: Supplementary file 1 [file ijms-24-10087-s001.zip › Supplementary Figure S1_participants flowchart_new.tif]
